# Characterization of the Trans-Epithelial Transport of Green Tea (*C. sinensis*) Catechin Extracts with In Vitro Inhibitory Effect against the SARS-CoV-2 Papain-like Protease Activity

**DOI:** 10.3390/molecules26216744

**Published:** 2021-11-08

**Authors:** Carmela Maria Montone, Sara Elsa Aita, Anna Arnoldi, Anna Laura Capriotti, Chiara Cavaliere, Andrea Cerrato, Carmen Lammi, Susy Piovesana, Giulia Ranaldi, Aldo Laganà

**Affiliations:** 1 Dipartimento di Chimica, Università di Roma La Sapienza, Piazzale Aldo Moro 5, 00185 Roma, Italy; carmelamaria.montone@uniroma1.it (C.M.M.); saraelsa.aita@uniroma1.it (S.E.A.); chiara.cavaliere@uniroma1.it (C.C.); andrea.cerrato@uniroma1.it (A.C.); susy.piovesana@uniroma1.it (S.P.); aldo.lagana@uniroma1.it (A.L.); 2 Dipartimento di Scienze Farmaceutiche, Università degli Studi di Milano La Statale, Via Mangiagalli, 25, 20133 Milano, Italy; anna.arnoldi@unimi.it; 3 CREA, Food and Nutrition Research Centre, 00100 Rome, Italy; giulia.ranaldi@crea.gov.it; 4 CNR NANOTEC, Campus Ecotekne, University of Salento, Via Monteroni, 73100 Lecce, Italy

**Keywords:** Caco-2 cells, catechins, COVID-19, polyphenols, high-resolution mass spectrometry, in vitro protease assay, *C. sinensis*

## Abstract

This work describes an untargeted analytical approach for the screening, identification, and characterization of the trans-epithelial transport of green tea (*Camellia sinensis*) catechin extracts with in vitro inhibitory effect against the SARS-CoV-2 papain-like protease (PLpro) activity. After specific catechin extraction, a chromatographic separation obtained six fractions were carried out. The fractions were assessed in vitro against the PLpro target. Fraction 5 showed the highest inhibitory activity against the SARS-CoV-2 PLpro (IC_50_ of 0.125 μg mL^−1^). The untargeted characterization revealed that (−)-epicatechin-3-gallate (ECG) was the most abundant compound in the fraction and the primary molecule absorbed by differentiated Caco-2 cells. Results indicated that fraction 5 was approximately 10 times more active than ECG (IC_50_ value equal to 11.62 ± 0.47 μg mL^−1^) to inhibit the PLpro target. Overall, our findings highlight the synergistic effects of the various components of the crude extract compared to isolated ECG.

## 1. Introduction

COVID-19 is a viral disease caused by SARS-CoV-2, a new strain of single-stranded RNA viruses of the Coronavirus (CoV) family, which is responsible for the current pandemic outbreak [1,2].

The pandemic emergency prompted the scientific community to pursue efforts for developing pharmacological therapies through the screening of some old drugs capable of working against SARS-CoV-2 and vaccines for counteracting this new disease threat [3,4]. In this context, identifying natural compounds able to prevent infection represents an efficient and complementary strategy. Indeed, many bioactive substances, which are naturally present in foods, have widely displayed potent biological activity [5,6]. Therefore, natural substances, especially those within plant-derived phytocomplexes, may represent sources of active compounds that may synergistically impair the SARS-CoV-2 infection and COVID-19 progression [7,8].

Catechins, a class of phenolic compounds, primarily present in food products, such as cocoa, red wine, fruits, vegetables, and tea leaves, are already well known for their interesting health-promoting activities, including anti-inflammatory and antioxidant antibacterial, anticancer, and neuroprotective ones [9]. In this field, green tea (*C. sinensis*) is one of the wealthiest catechin sources that contains (−)-epicatechin (EC), (−)-epicatechin-3-gallate (ECG), (−)-epigallocatechin (EGC), and (−)-epigallocatechin-3-gallate (EGCG) as its major catechin components. These molecules showed the ability to eradicate infectious agents and prevent infections [10]. In fact, several studies highlighted that EGCG can inhibit influenza virus replication in cell culture and that catechins show direct viricidal effects against various viruses, such as *Retroviridae*, *Orthomyxoviridae*, and *Flaviviridae*. In addition, EGCG interferes with the human immunodeficiency virus (HIV), blocking the enzymatic activity of the HIV-1 reverse transcriptase, hepatitis C virus (HCV), and herpes simplex virus 1 and 2 (HSV-1 and HSV-2) [11,12,13]. A potential mechanism underlying the antiviral effect seems to be related to the ability of flavonoids to inhibit the SARS-CoV-2 proteins that are considered to be the best drug targets, i.e., the main protease Mpro or 3-Chymotrypsin-like protease (3CLpro), papain-like protease (PLpro) [14], the spike protein (S), and the RNA-dependent RNA polymerase (RdRp) [15]. Interestingly, molecular-docking studies elucidated the binding affinities and binding mode between ECGC and Mpro targets [16]. Together with this mechanism of action, the inactivation of the virus and the inhibition of the replication were also considered [17,18,19].

On the other hand, it is well known that plant-derived phytocomplexes may be more active than the single main components within the extract since synergistic and/or additive mechanisms of action may concomitantly occur [20]. In addition, the improved bioavailability and the diversity of biological activities of the different extract components make the phytocomplex more advantageous. Nevertheless, the physiological activity of plant-derived phytocomplexes depends on their bioavailability, which is a challenging issue. In general, the intestinal transport of polyphenols is potentially influenced by several affecting factors, such as food matrix, biotransformation, and conjugation occurring during absorption [21]. Up to now, many studies investigated the absorption of single catechins, which is usually poor [22,23]. It was suggested that catechins, which are small, water-soluble molecules, are absorbed via the paracellular pathway in the small intestine [24].

Taking into account all these observations and the framework of research aimed at fostering the health-promoting activity of the green tea phytocomplex against COVID-19, the objective of the present study was the development of an analytical platform for the extraction, separation, and comprehensive identification of the most active fractions of a green tea polyphenol extract. Basically, after a specific solvent extraction for catechins, a chromatographic separation, based on reversed-phase liquid chromatography (RP-LC) was carried out for obtaining six fractions (F1-F6) which inhibitory activity was assessed in vitro against the PLpro target. The most active fraction (F5) was further analyzed by high-resolution mass spectrometry (HRMS) to annotate its composition. Finally, since little is known about the absorption and interaction of catechins within a phytocomplex whose complex composition may interact and modulate the transport, using Caco-2 cells as a model, the potential for intestinal transportation of relevant catechins was investigated.

## 2. Result and Discussion

### 2.1. In Vitro Inhibitory Effects of Green Tea Fractions on the PLpro Activity and Identification of Phenolic Compounds in the Most Active Fraction by Untargeted Approach

**Figure 1:**
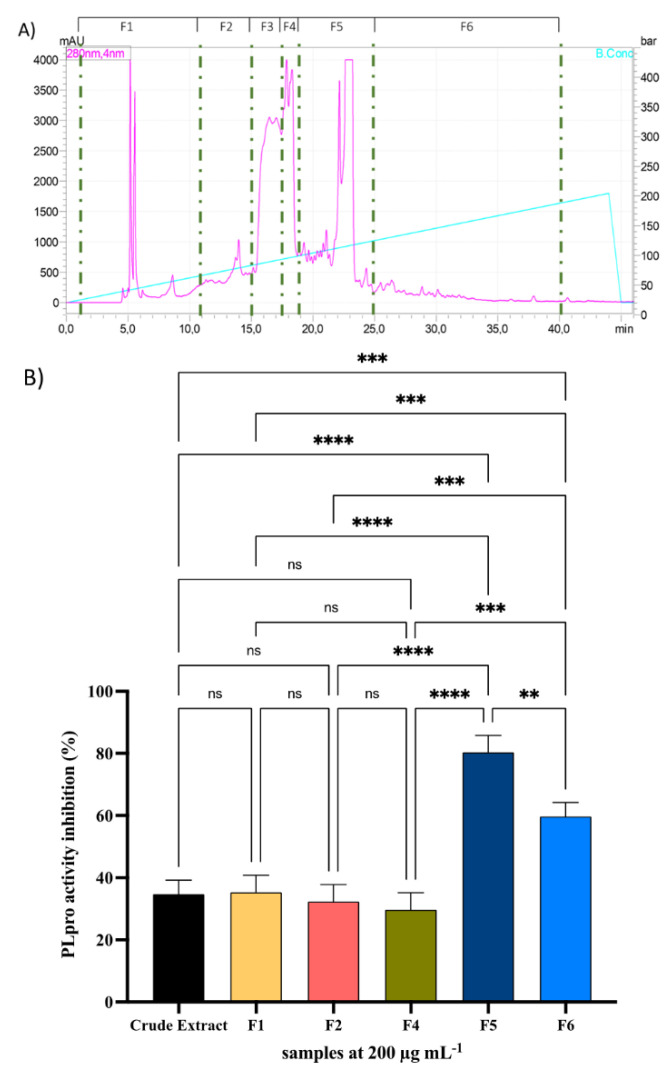
Chromatographic conditions for the fractionation of flavonoids in green tea extract (**A**) PLpro inhibitory effect of the crude extract and fractions F1, F2, F4, F5, and F6. (**B**) Bars represent the mean ± s.d. of three independent experiments performed in triplicate. Data were analyzed by one-way ANOVA followed by Tukey post hoc test. ns: not significant, ** *p* < 0.1, *** *p* < 0.001; **** *p* < 0.0001.

A dedicated protocol was applied for catechin extraction to understand whether green tea extracts might inhibit the PLpro activity of SARS-CoV-2. Hence, the total extract was fractionated by RP chromatography, obtaining six fractions (F1–F6); their inhibitory activity against PLpro was assayed (Figure 1A,B). Experiments were carried out in parallel using the crude extract. Results suggested that both the crude extract and all the six fractions (F1–F6) inhibit PLpro at the fixed concentration of 200 µg mL^−1^ (Figure 1B). In particular, F5 was the most active one, showing an inhibitory activity of 80.3 ± 5.5% at 200 µg mL^−1^, whereas the other fractions inhibited the PLpro by 35.3 ± 5.5%, 32.3 ± 3.3%, 29.7 ± 5.5%, and 59.7 ± 4.5% for F1, F2, F4, and F6, respectively. The F3 was discarded because it contained mainly caffeine, a molecule belonging to the methylxanthine class. Our study aimed to analyze phenolic compounds, and the presence of a highly concentrated molecule, such as caffeine, could distort our results.

Results indicated that the crude extract inhibited the PLpro activity by 34.7 ± 4.5%, suggesting a potency similar to F1, F2, and F4 (Figure 1B).

On the contrary, the fractionation process promoted the reduction of sample complexity and the modulation of the most active compound profile present in green tea extract. In particular, the fractionation process was effective for F5 and F6, which showed the target’s better inhibitory activity. From a statistical point of view, F5 and F6 are the most active fractions compared to crude extract and all the other fractions.

**Figure 2:**
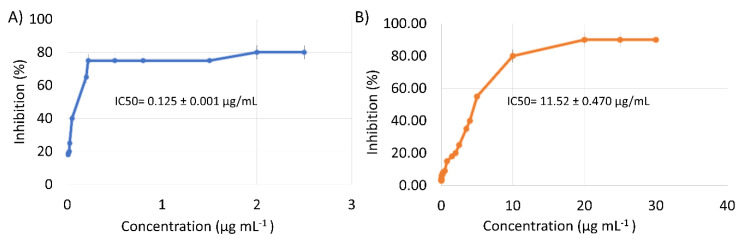
Dose-response curve of fraction F5 (**A**) and of the EGC standard (**B**).

Considering that F5 was more active than F6 (*p* < 0.1), a deeper investigation of its in vitro effect on the PLpro activity was carried out. Our findings indicated that F5 inhibited the PLpro with a dose-response trend and an IC_50_ value equal to 0.13 ± 0.001 µg mL^−1^ (Figure 2A).

Based on these results, the phenolic composition of fraction F5 was comprehensively characterized by UHPLC coupled to a Q Exactive Orbitrap mass spectrometer by a suspect screening data processing, based on a methodology already developed and implemented in the Compound Discoverer software by our research group [25].

Because of the wide range of bond energies in phenolic compound structures (from the weak acetal to the strong aromatic bonds), the acquisition was performed with a three-stepped NCE 20-40-60 for negative ion mode. In Table S2 (Supplementary materials), the annotated flavonoids were reported alongside some details, i.e., retention time, proposed formula, experimental *m*/*z*, accuracy, primary diagnostic product ions, and confidence level in ESI(−).

Forty-five flavonoids were tentatively identified in the F5 fraction. The most abundant annotated compound was ECG with 56.6% of the total flavonoid peak, followed by a minor amount of kaempferol (5.7%), EGCG (4.4%), and quercetin (3.8%). Our results agreed with previous findings on green tea [26,27].

In light of the F5 fraction composition and to assess whether this fraction’s antiviral activity might be mainly ascribed to the presence of ECG, biological experiments were performed using an authentic sample of this phytochemical. Figure 2B shows that the ECG standard dropped the target activity with a dose-response behavior and a calculated IC_50_ value equal to 11.62 ± 0.47 μg mL^−1^. Therefore, F5 was approximately 10 times more active than ECG. This different potency may be explained considering that the synergistic effect of all molecules in the isolated fraction, even in the low abundances, may positively contribute to the enhanced inhibitory activity of the PLpro target. A recent molecular docking study highlighted that ECG, kaempferol, ECGC, and quercetin may directly bind PLpro, impairing its activity [28,29]. Notably, in silico studies suggested that ECG possesses a higher binding affinity towards PLpro than other catechins [30]. More in detail, it was demonstrated that ECG binds to the S1 ubiquitin-binding site of PLpro; this may be owing to the phenolic hydroxyl group of the ECG, which can interact more easily with the heteroatoms of the amino acids of PLpro, in particular Gly-266 and Gln-269, through H-bonds. Moreover, ECG could form hydrophobic interactions with Met-208, Pro-247, Pro-248, Tyr-264, and Tyr-273 residues [31]. These shreds of evidence highlighted the importance of phytocomplexes. Indeed, the presence of more than a single molecule may influence the absorption, stability, and release of certain active compounds. For this reason, it was decided to carry out a trans-epithelial transport experiment on fraction F5, using differentiated Caco-2 cells.

### 2.2. Trans-Epithelial Transport of Fraction F5 by Differentiated Caco-2 Cells

The trans-epithelial transport of the F5 phytocomplex was investigated using differentiated Caco-2 cells. Notably, Caco-2 cells are a valuable and reliable model to rapidly assess the cellular permeability of potentially bioactive compounds, elucidate pathways of their transport, and study their metabolism in the gut [32]. This model, initially developed for drug-absorption screening, is also successfully applied worldwide to study food bioactivity [33]. The steady-state study was designed to treat Caco-2 cells with the fraction F5 at 20, 50, and 200 µg mL^−1^ for 2 h. The treatment at 50 and 200 µg mL^−1^ affected the monolayer integrity as monitored by TEER (transepithelial electrical resistance) values and phenol red passage (data not shown). In contrast, the treatment at the lowest concentration (20 µg mL^−1^) did not modify the integrity of cellular monolayer permeability. Thus, the trans-epithelial transport study was carried out at this condition. After 2 h of incubation, the AP and BL media were collected from each filter and desalted, dried, and re-dissolved in a suitable solution to allow their analysis by UHPLC coupled with HRMS. The phenols recovered in the AP gave some information on the stability of the F5 extract components after incubation with the brush border of intestinal cells. Instead, the phenols recovered in the BL side provided information on their transport by mature Caco-2 cells. Overall, the AP samples showed a composition similar to that of the F5 extract in terms of peak area intensity (Supplementary materials Table S2). The MS identifications revealed the presence of the same compounds in both samples and confirmed that similar to the total extract, the most abundant compound in the AP sample is the ECG (see Table S2). The peak intensity area of each identified compound is lower in AP samples than in the F5 extract, suggesting that phenolic compounds may be uptaken by the differentiated Caco-2 cells or metabolized. In particular, it is known that Caco-2 cells express human phase I and phase II metabolizing enzymes [34]. It was demonstrated that ECG, characterized by poor absorbability, may undergo metabolic changes when tested alone in the presence of the AP enzyme of differentiated Caco-2 cells. For ECG, three marginal amounts of metabolites were detected in a previous paper [35], i.e., methylated ECG, sulphate conjugate of ECG, and methylated sulphate conjugate of ECG. Based on these considerations, our AP samples were assessed to precisely monitor these metabolites. Our results suggest that when tested within the phytocomplex, ECG did not undergo these metabolite productions. Of course, we cannot exclude that they were produced in amounts that remained below the detection and quantification values limit. The obtained data suggest a selective transport of only ECG of the F5 phenols by Caco-2 cells into the BL compartment. The other most abundant annotated flavonoids, i.e., kaempferol and quercetin, were not flawed or transported, probably due to their poor water solubility. Even if quercetin has potent antioxidant, anti-inflammatory, immunomodulatory, and antiviral properties and a very high safety profile, as with most other polyphenols, it shows a meager rate of oral absorption, and its clinical use is considered by most of modest utility [36].

## 3. Materials and Methods

### 3.1. Chemicals and Materials

All chemicals, reagents, and organic solvents of the highest grade available were purchased from Merck (St. Louis, MO, USA) unless otherwise stated. LC-MS grade water, methanol (CH_3_OH), and acetonitrile (ACN) were purchased from Thermo Fisher Scientific (Waltham, MA, USA). The tea powder was bought in a local supermarket.

### 3.2. Catechins Extraction

A 0.4 g green tea (*C. sinensis*) leaf powder sample was extracted with 8 mL of a 50% ethanol solution. The mixture was sonicated at room temperature (RT) for 1 h, and the solution was centrifuged for 30 min (4000 rpm, 25 °C). The supernatant was collected, and the procedure was repeated once. The two supernatants were pooled and evaporated by an IKA RV 8 Rotary Evaporator (IKA-Werke GmbH & Co. KG, Staufen, Germany) up to a volume of 0.5 mL. The sample was filtered through a 0.22 μm membrane filter and stored at −20 °C until use.

### 3.3. Phenolic Extract Fractionation

Phenolic compounds were purified using a column Xbridge^®^ BEH C18 (4.6 mm × 250 mm, 130 Å, 5µm particle size, Waters, Milford, MA, USA). The column was connected to the Shimadzu Prominence LC-20AD system, including a CBM-20A controller, two LC-20 AD XR pumps, and a DGU-20As online degasser, equipped with an SPD-M20A UV detector, and an autocollector FRC-10A (Shimadzu) was employed. Data acquisition was performed by the LabSolution version 5.53 software (Shimadzu, Kyoto, Japan). The detector was set at 280 nm. The sample was eluted at a flow rate of 0.6 mL min^−1^ using ddH_2_O with 10 mmol L^−1^ ammonium formate at pH 10 as phase A and MeOH:ddH_2_O (90:10, *v*/*v*) with 10 mmol L^−1^ ammonium formate at pH 10 as phase B. The gradient started at 5% of B, then increased to 50% in 44 min; then, the column was equilibrated for 10 min. Six fractions were collected as follows: F1 (1–11 min), F2 (12–14 min), F3 (15–16 min), F4 (16–19 min), F5 (20–25 min), and F6 (26–40 min). The number of collected fractions from chromatographic separation is shown in Figure 1A.

Each collected fraction (F1–6) was subjected to a PLpro bioactivity test to identify the most active ones.

### 3.4. Quantification of Total Phenolic Content

The determination of the total phenolic content in the green tea leaves crude extract and the six collected fractions was carried out using the BCA assay according to the Pierce™ BCA Protein Assay Kit manufacturer’s instructions with some modifications previously described [37]. Briefly, gallic acid was used as the standard for the calibration curve (range 0.025–0.5 mg mL^−1^). The fraction volumes were normalized before analysis.

Total phenolic content was expressed as milligrams of gallic acid equivalents (GAE) per solution volume of the sample. Details are reported in Supporting Information Table S1.

### 3.5. In Vitro Inhibition Assay of PLpro Activity and Calculation of IC_50_ Values

A Papain-like Protease (SARS-CoV-2) Assay Kit (BPS Bioscience, https://bpsbioscience.com) was used to test the inhibitory activity of the total extract and the six collected fractions (F1–F6). The steps of in vitro assay were carried out following manufacturer’s protocol. In brief, each reaction was completed in a 50 µL volume in 96-well plates. The samples were reconstituted in H_2_O. Each reaction solution contained 15 ng recombinant PLpro (the final concentration in the reaction was 0.3 ng µL^−1^), 1.0 mmol L^−1^ DTT, and 5 mmol L^−1^ fluorogenic substrates (blank sample). GRL0617 (10 μmol L^−1^) was used as inhibitor control.

The reaction was started by adding 10 μL of the substrate solution to each well (final concentration of the PLpro substrate in a 50 μL reaction was 50 μM).

The reaction mixtures were incubated at 37 °C for 60 min. The fluorescence intensity of each reaction was measured and recorded on a microtiter plate-reading fluorimeter (GloMax^®^ Discover, Promega, Madison, Wisconsin, USA). The excitation wavelength was 360 nm, and the detection emission wavelength was 460 nm. The inhibition activity (IA) was calculated by the equation:

$$IA\;\left(\%\right)=\frac{\left(I_{F,BLANK}-I_{F,\;SAMPLE}\right)}{\left(I_{F,BLANK}-I_{F,CTRL}\right)}\times 100$$

where *I_F_*_,*BLANK*_ was the fluorescence intensity of blank sample (PLpro, DTT, and fluorogenic substrate), *I_F__,SAMPLE_* was the fluorescence intensity of the extracted green tea fraction sample, and *I_F,CTRL_* was the fluorescence intensity of the inhibitor control (GRL0617).

### 3.6. UHPLC-MS/MS Analysis

The most active fraction was analyzed by UHPLC coupled with HRMS. The chromatographic separation of the phenolic compounds was carried out using a Vanquish Binary Pump H (Thermo Fisher Scientific, Bremen, Germany), equipped with a thermostated autosampler and column compartment, on a Kinetex core-shell C18 column (100 mm × 2.1 mm i.d.) with a particle size of 2.6 µm (Phenomenex, Torrance, CA, USA) at 40 °C and with a flow rate of 0.6 mL min^−1^. The injection volume was 10 µL. The mobile phases consisted of H_2_O/HCOOH (99.9:0.1, *v*/*v*; phase A) and ACN/HCOOH (99.9:0.1, *v*/*v*; phase B). The elution gradient and ESI source parameters were optimized in our previous studies [25,38]. The chromatographic system was coupled to a Q Exactive Hybrid Quadrupole-Orbitrap Mass Spectrometer (Thermo Fisher Scientific). Samples were analyzed in TOP 5 data-dependent acquisition (DDA) mode with an exclusion list containing the most intense ions detected in the blank sample (H_2_O/MeOH, 90:10, *v/v*). For low- and high-molecular-weight phenolic compound analysis, MS data were acquired in the range 150–1000 *m*/*z* and 300–2000 *m*/*z*, respectively, with a resolution (full width at half maximum, FWHM, at *m*/*z* 200) of 70,000. In full-scan mode, the automatic gain control (AGC) target value was 200,000, the maximum ion injection time was 100 ms, and the isolation window width was 2 *m*/*z*. Tandem MS (MS/MS) fragmentation was performed with a resolution (FWHM, at *m/z* 200) of 35,000 with the AGC target value set at 100,000, and dynamic exclusion set to 3 s. Fragmentation was achieved in the higher-collision dissociation (HCD) cell at three values of normalized collision energy (NCE), namely, 20-50-80 NCE in the positive ion mode and 20-40-60 NCE in the negative ion mode based on the results of a previous study [25]. All samples were run in triplicate.

### 3.7. Data Analysis and Phenolic Compounds Validation

For phenolic compound annotation, a customized data-processing workflow on Compound Discoverer 3.1 (Thermo Fisher Scientific, Waltham, MA, USA) was employed [25,39]. A metabolomics-based approach was chosen, aided by a customized phenolic compound database, which was generated by combining free phenolic compounds (aglycones) with a series of sugars and aliphatic and aromatic acids. The database, complete with IDs, accurate masses, and molecular formulas, was implemented in the mass list feature to automatically match extracted *m*/*z* ratios (45,567 combinations). For simplifying MS/MS spectra manual annotation, detailed HCD fragmentation spectra for flavonoids and phenolic acids were implemented in the compound class scoring node. Moreover, the parameters for the predict composition tool were adapted to phenolic compounds. Extracted *m*/*z* from the raw chromatograms were grouped, aligned, and filtered to remove background compounds and features not associated with compounds present in the databases or with MS/MS spectra. Filtered compounds were manually validated by matching fragmentation spectra to available standards or spectra reported in the literature. When data were lacking, phenolic compounds were tentatively identified according to the characteristic fragmentation spectra.

### 3.8. Caco-2 Cell Culture and Differentiation

Caco-2 cells were kindly obtained from Institut National de la Santé et de la Recherche Médicale (INSERM, Paris). For differentiation, Caco-2 cells were seeded on polycarbonate filters, 12 mm diameter, 0.4 µm pore diameter (Transwell, Corning Inc., Lowell, MA, USA) at a density of 3.5 × 105 cells/cm^2^ in complete medium supplemented with 10% FBS in both apical (AP) and basolateral (BL) compartments for 2 d to allow the formation of a confluent cell monolayer. Starting from day three after seeding, cells were transferred to a FBS-free medium in both compartments, supplemented with ITS (final concentration 10 mg/L insulin (I), 5.5 mg/L transferrin (T), 6.7 μg/L sodium selenite (S); GIBCO-Invitrogen, San Giuliano Milanese, Italy) only in the BL compartment, and allowed to differentiate for 21 days with regular medium changes three times weekly [40].

### 3.9. Cell Monolayers Integrity Evaluation

The transepithelial electrical resistance (TEER) of differentiated Caco-2 cells was measured at 37 °C using the voltmeter apparatus Millicell (Millipore Co., Burlington, MA, USA) immediately before and at the end of the absorption experiments. In addition, at the end of the absorption experiments, cells were incubated from the AP side with 1 mM phenol red in PBS with CaCl_2_ and MgCl_2_ for 1 h at 37 °C to monitor the paracellular permeability of the cell monolayer. The BL solutions were then collected, and NaOH (70 µL, 0.1 N) was added before reading the absorbance at 560 nm in a microplate reader (Synergy H1, Biotek, Winooski, VT, USA). The phenol red passage was quantified using a standard phenol red curve. Only filters showing TEER values and phenol red passages similar to untreated control cells were considered for sample transport analysis.

### 3.10. Trans-Epithelial Transport of F5 Extract

Before the experiments, the cell monolayer integrity and differentiation were checked by TEER measurement as described above. Cells were then washed twice, and sample (F5) trans-epithelial transport was assayed. Transport experiments were performed in transport buffer solution (137 mM NaCl, 5.36 mM KCl, 1.26 mM CaCl_2_, and 1.1 mM MgCl_2_, 5.5 mM glucose) according to previously described [40]. The most active fraction absorption and the metabolism were assayed by loading the upper compartment with the most active fraction (at the concentration of 200, 50, and 20 μg mL^−1^) in the AP transport solution (400 µL) and the lower compartment with the BL transport solution (600 µL). Transport experiments were conducted for 2 h. In particular, the plates were incubated at 37 °C, and the BL solutions were collected after 2 h. All BL solutions, together with the AP solutions collected at the end of the transport experiment, were stored at −80 °C before analysis. Two independent absorption experiments were performed, each in duplicate.

### 3.11. Statistical Analysis

Statistical analyses were carried out by one-way ANOVA followed by Tukey test (GraphPad Prism 9, Jolla, San Diego, CA, USA). Values were expressed as means ± s.d.; *p*-values < 0.05 were considered to be significant.

## 4. Conclusions

This study describes an effective multidisciplinary strategy for mining a fraction of green tea that can inhibit the activity of SARS-CoV-2 PLpro. To foster the antiviral effect of this fraction, this study provides new pieces of evidence regarding its composition and the trans-epithelial transport of the main components of the phytocomplex. Findings indicate that the investigation of the antiviral properties of these polyphenols against SARS-CoV-2 may represent an additional tool to be used in co-treatment with conventional antiviral drugs to control this ongoing pandemic.

Hence, it would be valuable to examine the effect of green tea on the spread of SARS-CoV-2 in vivo. In addition, further clinical trials will be required to reveal the impact of tea consumption on COVID-19 prognosis.

## Data Availability

Table S1. Total phenolic content (TPC) measured for the six purified fractions (F1–F6); Table S2. List of tentatively identified compounds.

## Supplementary Materials

The following are available online, Table S1: Total phenolic content (TPC) measured for the six purified fractions (F1–F6); Table S2: List of tentatively identified compounds.

## Sample Availability

Samples of the compounds not are available from the authors.

## Abbreviations

ACN	acetonitrile
AGC	automatic gain control
AP	apical
BL	basolateral
3CLpro	3-chymotrypsin-like protease
CoV	Coronavirus
DDA	data-dependent acquisition
EC	(-)-epicatechin EC
ECG	(-)-epicatechin-3-gallate (ECG)
EGC	(-)-epigallocatechin (EGC)
EGCG	(-)-epigallocatechin-3-gallate (EGCG)
ESI	electrospray ionization
FWHM	full width at half maximum
GAE	gallic acid equivalent
HCD	higher energy collision dissociation
IA	inhibition activity
HRMS	high-resolution mass spectrometry
MeOH	methanol
MRS	De Man, Rogosa, and Sharpe
MS	mass spectrometry
NCE	normalized collision energy
PLpro	papain-like protease
RT	room temperature
RP-LC	reversed-phase liquid chromatography
S	spike protein
