# Supplementary material for: Characterization of the Trans-Epithelial Transport of Green Tea (C. sinensis) Catechin Extracts with In Vitro Inhibitory Effect against the SARS-CoV-2 Papain-like Protease Activity
Source: Molecules. 2021 Nov 8;26(21):6744. doi: 10.3390/molecules26216744 (PMC8587865; doi:10.3390/molecules26216744)
Supplement: Supplementary file 1 [file molecules-26-06744-s001.zip › molecules-1418045-supplementary/Supplementary Material/Supporting information for review only.pdf]

Supporting information for review only

The pandemic emergency prompted the scientific community to pursue efforts for developing pharmacological therapies and vaccines for counteracting this new disease threat. In this context, the identification of natural compounds able to prevent infection represents an efficient and complementary strategy. Indeed, natural substances may represent sources of active compounds which may impair the SARS-CoV-2 infection and COVID19 disease progression. Being aware that plant-derived phytocomplexes may be more active than the single main components within the extract since synergistic and/or additive mechanisms of action may concomitantly occur, the present study was aimed at fostering the health-promoting activity of the green tea phytocomplex against COVID-19 disease.

Indeed, for the first time, we provide new insights regarding the extraction, separation, and comprehensive identification of the most active fractions of a green tea phenolic extract. Overall, after a specific solvent extraction for catechins, a chromatographic separation, based on reversed-phase liquid chromatography (RP-LC), permitted us to obtain six fractions (F1-F6) whose inhibitory activities were assessed in vitro against the PLpro target. The most active fraction (F5) was further analyzed by high-resolution mass spectrometry to annotate its composition.

In addition, since little is known about the absorption and interaction of catechins when they are within a phytocomplex whose complex composition may interact and modulate the transport, the F5's phytocomplex bioavailability was evaluated performing an intestinal transport experiment using differentiated human intestinal Caco-2 cells. Our Findings clearly indicate that the investigation of the antiviral properties of these polyphenols against SARS-CoV-2 may represent an additional tool to be used in co-treatment with conventional antiviral drugs to control this dramatic pandemic.
