# Supplementary material for: Characterization of the Trans-Epithelial Transport of Green Tea (C. sinensis) Catechin Extracts with In Vitro Inhibitory Effect against the SARS-CoV-2 Papain-like Protease Activity
Source: Molecules. 2021 Nov 8;26(21):6744. doi: 10.3390/molecules26216744 (PMC8587865; doi:10.3390/molecules26216744)
Supplement: Supplementary file 1 [file molecules-26-06744-s001.zip › molecules-1418045-supplementary/Supplementary Material/Table S1.pdf]

Supplementary Material

Untargeted analytical approach for the screening, identification, and characterization of the trans-epithelial transport of green tea (Camellia sinensis) catechin extracts with in vitro inhibitory effect against the SARS-Cov-2 papain-like protease activity

Carmela Maria Montone[1], Sara Elsa Aita[1], Anna Arnoldi[2], Anna Laura Capriotti[1]*, Chiara Cavaliere[1], Andrea Cerrato[1], Carmen Lammi[2]*, Susy Piovesana[1], Giulia Ranaldi[3], Aldo Laganà[1,4]

[1]Dipartimento di Chimica, Università di Roma La Sapienza, Piazzale Aldo Moro 5, 00185 Roma, Italy

[2]Dipartimento di Scienze Farmaceutiche, Università degli Studi di Milano La Statale, Via Mangiagalli, 25, 20133 Milano, Italy

[3]CREA, Food and Nutrition Research Centre, Rome, Italy

[4]CNR NANOTEC, Campus Ecotekne, University of Salento, Via Monteroni, 73100 Lecce, Italy

** These authors equally contributed to this work

***Corresponding author**: Anna Laura Capriotti

Dipartimento di Chimica, Università degli Studi di Roma La Sapienza

Piazzale Aldo Moro 5

00185 Rome, Italy

E-mail: annalaura.capriotti@uniroma1.it

T (+39) 06 4991 3945

***Co-Corresponding author**: Carmen Lammi

Dipartimento di Scienze farmaceutiche, Università degli Studi di Milano

Via Mangiagalli, 25

20133 Milan, Italy

E-mail: carmen.lammi@unimi.it

T (+39) 02503 19372

Content

Table S1. Total phenolic content (TPC) measured for the 6 purified fractions (F1-F6).

Table S1: Total phenolic content (TPC) measured for the 6 purified fractions (F1-F6) and the crude green tea extract. The TPC was calculated by the following equation:

$$y = -4247.9\,x^2 + 14093x + 113.06$$

$$R^2 = 0.9974$$

	**TPC ± sd (mg GAE/mL)**
Fraction1	5.0 ± 0.3
Fraction2	5.9 ± 0.2
Fraction3	0.7 ± 0.0
Fraction4	15.2 ± 0.1
Fraction5	4.5 ± 0.3
Fraction6	0.2 ± 0.0
Crude Extract	16.9 ± 0.5
